# Melatonin alleviates chilling stress in cucumber seedlings by up-regulation of *CsZat12* and modulation of polyamine and abscisic acid metabolism

**DOI:** 10.1038/s41598-017-05267-3

**Published:** 2017-07-10

**Authors:** Hailiang Zhao, Kai Zhang, Xiaoting Zhou, Linjie Xi, Yuping Wang, Hongjun Xu, Tonghua Pan, Zhirong Zou

**Affiliations:** 1 College of Horticulture, Northwest Agricultural & Forest University, Yangling, Shaanxi China; 2 College of Horticulture, Shanxi Agricultural & Forest University, Taigu, Shanxi China; 3 Key Laboratory of Protected Horticulture Engineering in Northwest, ministry of Agriculture, Yangling, Shaanxi China; 4 Department of Garden Engineering, Gansu Agriculture Technology College, Lanzhou, Gansu China

## Abstract

To obtain new insights into the mechanisms of the positive effects of exogenous melatonin applications to cucumber seedlings during chilling, we investigated its role in regulating photosynthesis, the transcription level of *csZat12* and the metabolism of polyamines (PAs) and of abscisic acid (ABA). The negative effects of chilling were clearly alleviated in cucumber seedlings by irrigation with 200 μM melatonin solution. This was evidenced by alleviation of the decline in net photosynthesis rate and also in electrolyte leakage in chilled plants. The reasons for this can be explained as follows. First, melatonin up-regulates *CsZat12*, an important stress-related gene. Second, melatonin increases the content of putrescine (Put) and spermidine (Spd) and stabilized spermine (Spm) by altering the activity of the PA metabolic enzymes. And, third, ABA is also involved in these effects as melatonin modulated the expression of the key ABA biosynthesis genes (*CsNCED1* and *CsNCED2*) and also the key ABA catabolism genes (*CsCYP707A1* and *CsCYP707A2*). This study provides new evidence suggesting melatonin mitigates chilling stress in cucumber by up-regulating the expression of *CsZat12* and by modulating the metabolism of PAs and ABA.

## Introduction

Cucumber is an important salad crop in several countries and must be grown year-round to meet market demand. However, cucumber’s susceptibility to chilling stress is a major factor impacting its cultivation in the cooler months. Hence, in many regions, cucumber must be grown in green houses in winter^1, 2^.

Under chilling conditions, the cell membrane undergoes a phase change from liquid crystalline to gel^3^. This phase change is associated with a reduction in fluidity and the membrane becomes more rigid^3^. In addition, overproduction of reactive oxygen species (ROS) under chilling conditions leads to lipid peroxidation, this further compromises membrane integrity and so results in solute leakage^4^. Chilling stress also reduces the activity of most enzymes involved in plant metabolism^5^. Reduced enzyme activity together with membrane damage leads to dysfunction of a number of biochemical reactions and physiological functions, including photosynthesis^1^. Exogenous substrates such as melatonin have been used widely to help plants cope better with environmental stresses - including chilling stress^6^.

Melatonin is a low molecular-weight indole amine molecule. It was first reported in 1995 in several plants such as tomato, banana, cucumber, beetroot, potato^7^, and later found to be present in most plants and in a range of organs^8^. With similar roles to indole acetic acid, melatonin acts as a growth promoter and a rooting agent^9^. Based on its antioxidant properties, melatonin also plays an important role in protecting plants against abiotic stresses, including UV radiation^10^, heavy metals^11^, extreme temperatures^12^, salinity^13^ and chilling^14^. Moreover, melatonin could modulate polyamines (PAs) levels in rat brains^15^. It has been suggested melatonin may modify PA metabolism in plants so as to enhance chilling tolerance. However, little is known about how melatonin modulates PA metabolism.

PAs are small aliphatic amines; the three most common ones found in plants are diamine putrescine (Put), triamine spermidine (Spd) and tetramine spermine (Spm)^16^. Owing to their positive charge at physiological values of pH, these PAs help to preserve the structure of nucleic acids and proteins, to maintain the integrity of membranes and to sustain enzyme activity. In this way they serve to protect the physical, biochemical functions of plants through their interactions with nucleic acids, proteins and phospholipids^17^. Melatonin may exert its protect effects through PAs.

As a C2H2 zinc finger transcription factor, *Zat12* regulates a serious of transcripts involved in plant acclimation to high light, wounding, chilling and oxidative stress^18^. The previous studies also show that *Zat12* loss-of-function plants are sensitive to stress while the tolerance can be recovered by overexpressing *Zat12* ^18–21^. Moreover, *Zat12* also up-regulates *ADC1*&*ADC2* involved in Put synthesis^22^.

It has been hypothesized that Put can cause the accumulation of the important plant hormone, abscisic acid (ABA) by activating zeaxanthin degradation^23, 24^. In addition to its involvement in plant growth and development, ABA is also centrally involved in a number of plant stress responses. Several stress-response genes can be up-regulated by ABA. Called ABA-responsive elements (ABRE) these are involved in many stress responses^25^. However, it is still unclear whether ABA is involved in the influence of melatonin on tolerance to chilling stress.

Till now, it has made great advance that melatonin could mitigate environment stress through its antioxidant activity^26^. Melatonin improves plant iron deficiency tolerance via increased accumulation of polyamine-mediated nitric oxide^27^. And melatonin can also induce cold tolerance via long distance signaling^28^ However, little is known about the other physiological functions of melatonin in relation to coping with chilling stress. Here, to obtain new insights into other stress-resistance mechanisms involving melatonin, we started by investigating the natural rhythms of PAs and ABA in cucumber, pre-treated with melatonin prior to exposure to chilling. We also explored how melatonin regulates PA and ABA metabolism. We studied the transcript level of *Zat12*; this gene is known to be important in relation to stress and also involved in Put synthesis through up-regulating ADC1&ADC2, and also responses to spermine^22^.

## Results

### Electrolyte leakage and net photosynthetic rate

**Figure 1:**
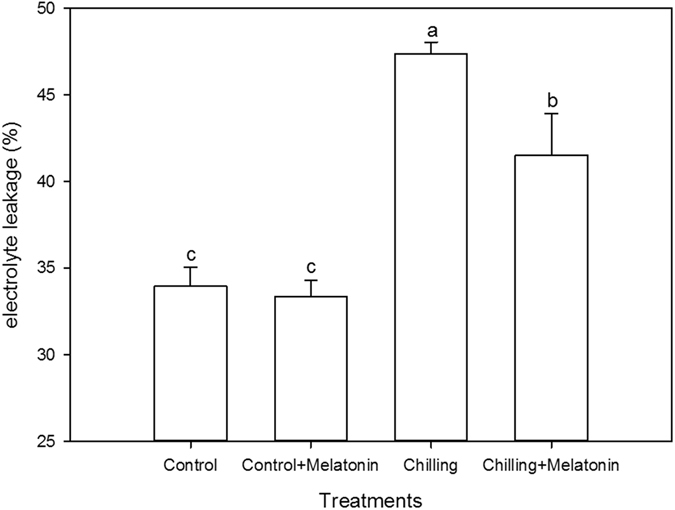
Effects of exogenous melatonin on electrolyte leakage after eight days chilling treatment. Control, treating at 28/18 °C; Control + Melatonin, pre-treating with 200 μM melatonin and treating at 28/18 °C; Chilling, treating at 15/8 °C; Chilling + Melatonin, pre-treating with 200 μM melatonin and treating at 15/8 °C; Date represent means ± SD of third replicate samples. Different letters indicate a significant difference in a particular series at P < 0.05 according to ANOVA and Duncan’s multiple range tests.

**Figure 2:**
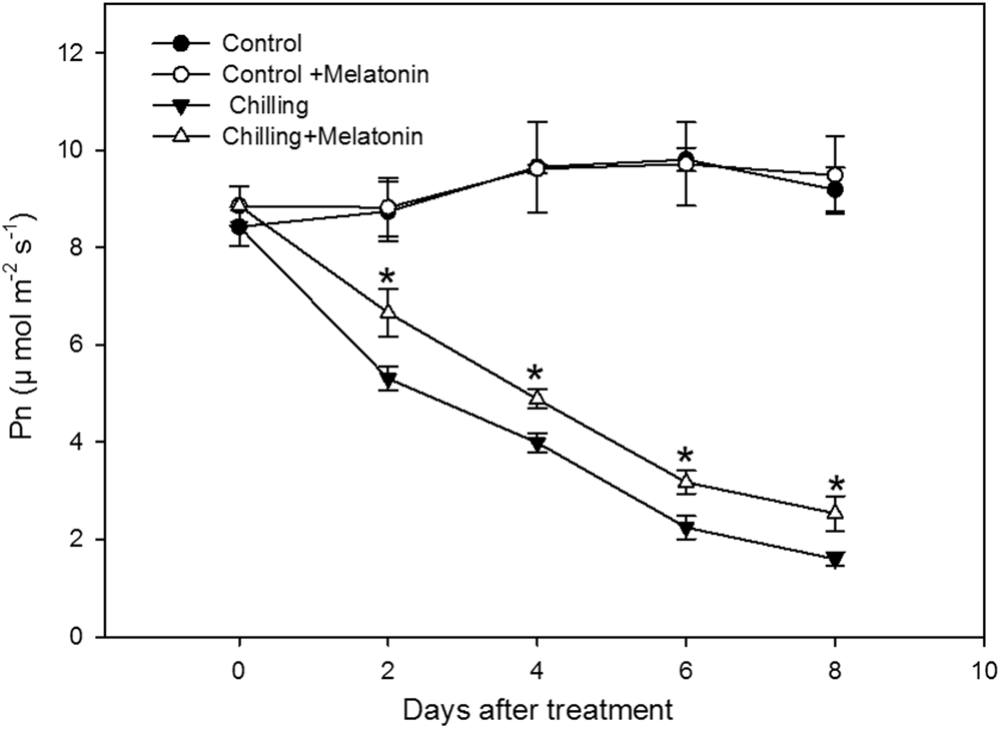
Effects of exogenous melatonin on net photosynthesis rate (Pn) under chilling stress. Control, treating at 28/18 °C; Control + Melatonin, pre-treating with 200 μM melatonin and treating at 28/18 °C; Chilling, treating at 15/8 °C; Chilling + Melatonin, pre-treating with 200 μM melatonin and treating at 15/8 °C; Date represent means ± SD of third replicate samples.

To examine whether melatonin mitigated chilling stress in cucumber seedlings, electrolyte leakage from leaves was measured. The electrolyte leakage of samples under chilling was 40% higher than in the un-chilled controls (Fig. 1). Melatonin pretreatment suppressed electrolyte leakage under chilling by 12%, compared with the non-melatonin treatment. We also monitored the net photosynthetic rate (Pn) during the experiment period. Under normal temperature conditions, Pn remained between 8.43 and 9.81 μmol·m^−2^·s^−1^ with slight fluctuations but with no significant difference between plants with and without melatonin pretreatment (Fig. 2). Under chilling conditions, Pn was significantly decreased. Also, the decline during the early period was greater than in the later period. Compared with the controls, Pn had dropped by 83% by day 8. However, melatonin pretreatment significantly mitigated this decline. Compared with the water pretreatment, the Pn of chilled seedlings with melatonin pretreatment was higher by 18 to 37%.

### PA level and PA metabolism enzyme activity

**Figure 3:**
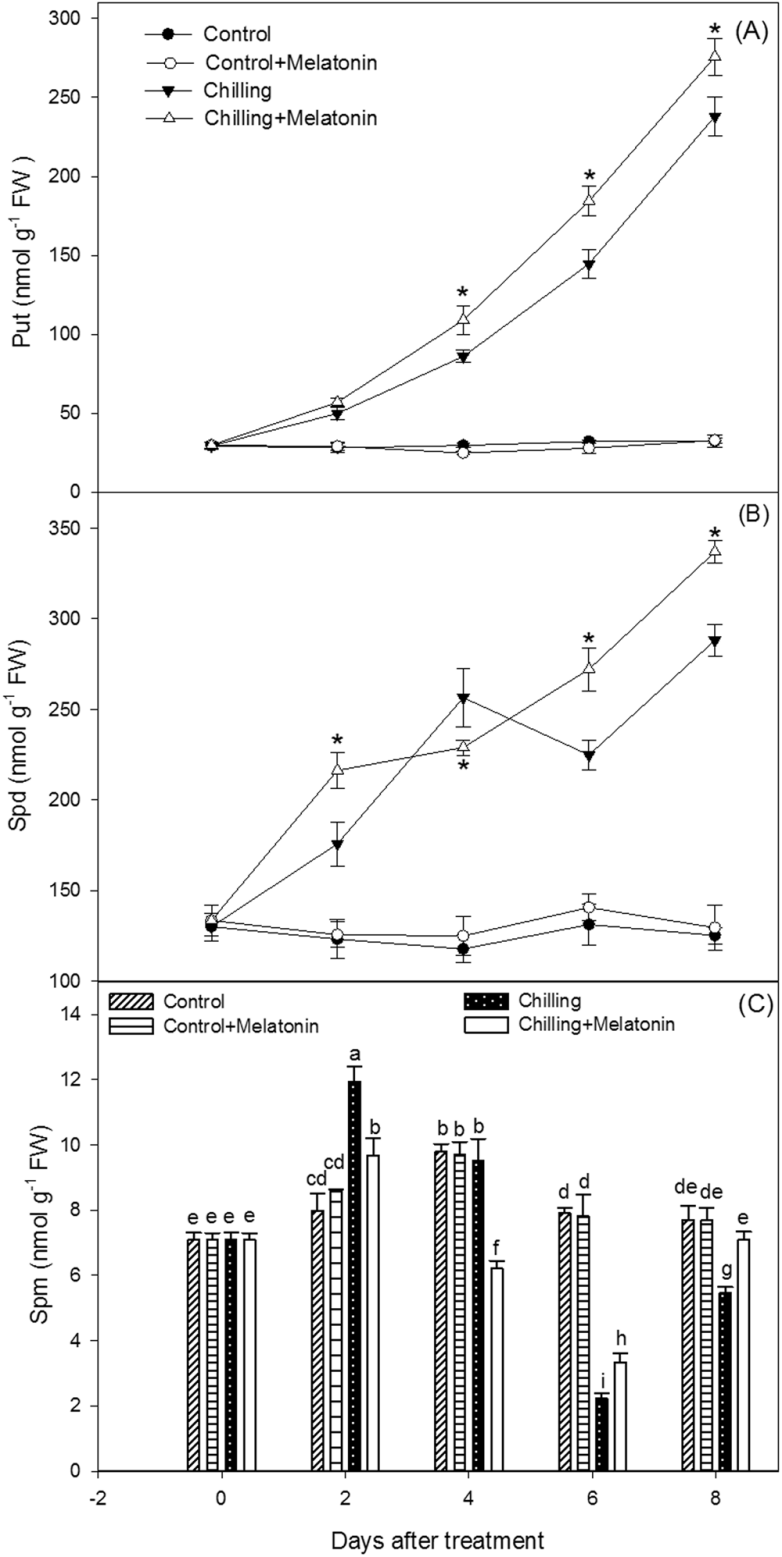
Effects of exogenous melatonin on polyamine contents under chilling stress. (**A**) Putrescine (Put), (**B**) spermidine (Spd), (**C**) spermine (Spm). Control, treating at 28/18 °C; Control + Melatonin, pre-treating with 200 μM melatonin and treating at 28/18 °C; Chilling, treating at 15/8 °C; Chilling + Melatonin, pre-treating with 200 μM melatonin and treating at 15/8 °C; Date represent means ± SD of third replicate samples, *Significant difference between different treatments at P < 0.05 based on Duncan’s multiple range test.

To determine whether PAs were involved in the ameliorating effects of melatonin, we monitored the levels of the PAs in leaves during the chilling period. The Put level rose dramatically under chilling (Fig. 3A). For example, by day 8 the Put content was 7.4-fold higher under chilling than in the controls. The melatonin pretreatment further increased Put content under chilling. Chilling also induced increase in Spd content (Fig. 3B). Under chilling, the Spd content had increased by 49% by day 4, it then declined slightly, before peaking at day 8. Melatonin pretreatment further increased the Spd content (except at day 4). Under chilling, the Spm content increased slightly by day 2, it then decreased over the next four days, before increasing again after day 6 when contents were similar to those in the controls (Fig. 3C). Melatonin to some extent mitigated the fluctuations in Spm, maintaining a slightly higher Spm content than for chilling alone (except at day 2).

**Figure 4:**
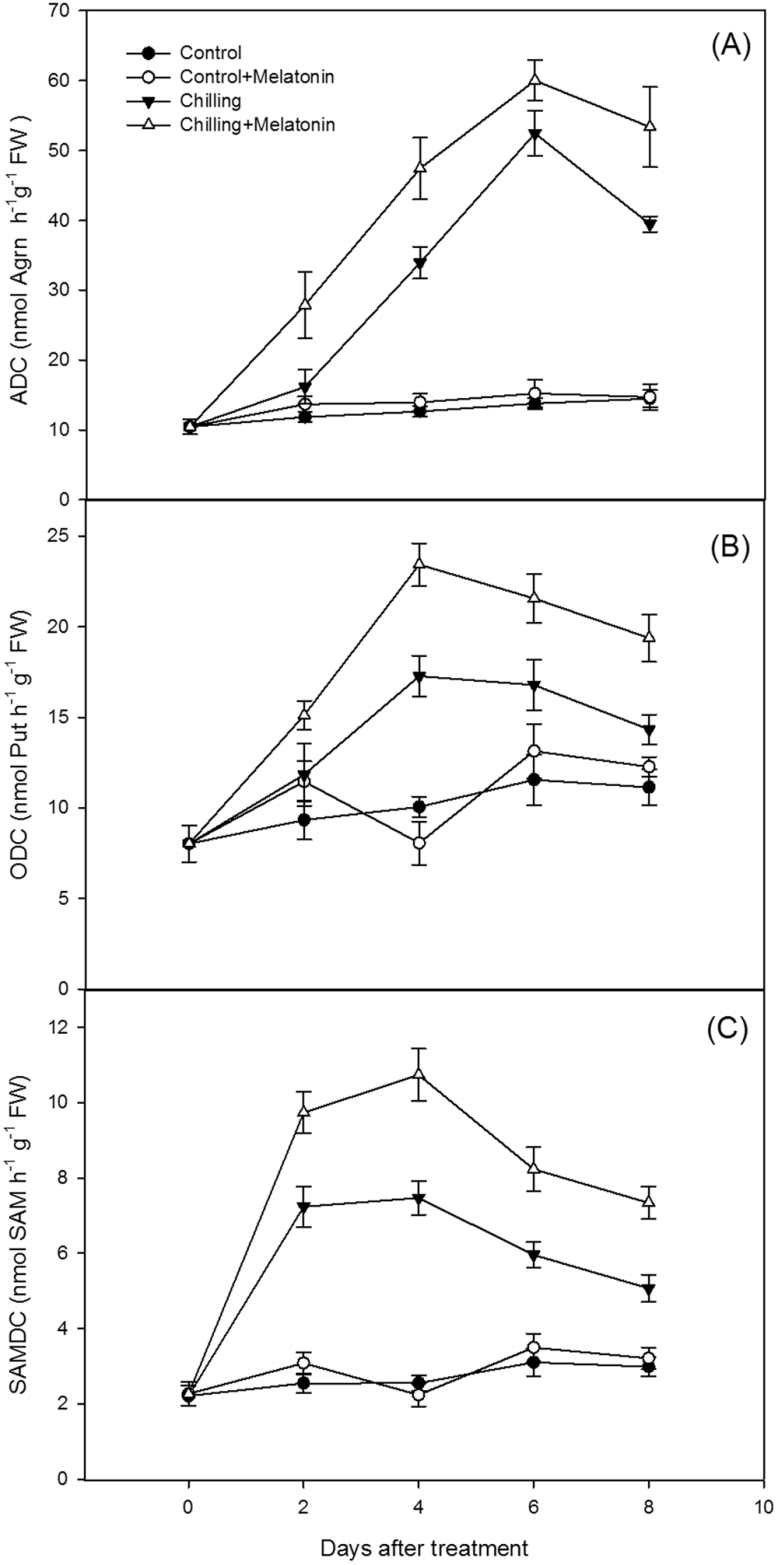
Effects of exogenous melatonin on polyamine biosynthesis enzyme activities under chilling stress. (**A**) Arginine decarboxylase (ADC), (**B**) ornithine decarboxylase (ODC), (**C**) S-adenosylmethionine decarboxylase (SAMDC). Control, treating at 28/18 °C; Control + Melatonin, pre-treating with 200 μM melatonin and treating at 28/18 °C; Chilling, treating at 15/8 °C; Chilling + Melatonin, pre-treating with 200 μM melatonin and treating at 15/8 °C; Date represent means ± SD of third replicate samples.

Under chilling, ADC activity showed no significant change over the first two days but then increased dramatically before peaking at day 6 (Fig. 4A). Melatonin accelerated the increase and significantly increased ADC activity. Chilling also increased ODC activity (Fig. 4B). This rose during the first four days before remaining high. Melatonin pretreatment did not significantly change this trend, but did increase the level significantly. Compared with the controls, chilling caused SAMDC activity to rise by day 2, but activity declined after day 4 (Fig. 4C). Melatonin pretreatment significantly reduced the later decline in SAMDC activity.

**Figure 5:**
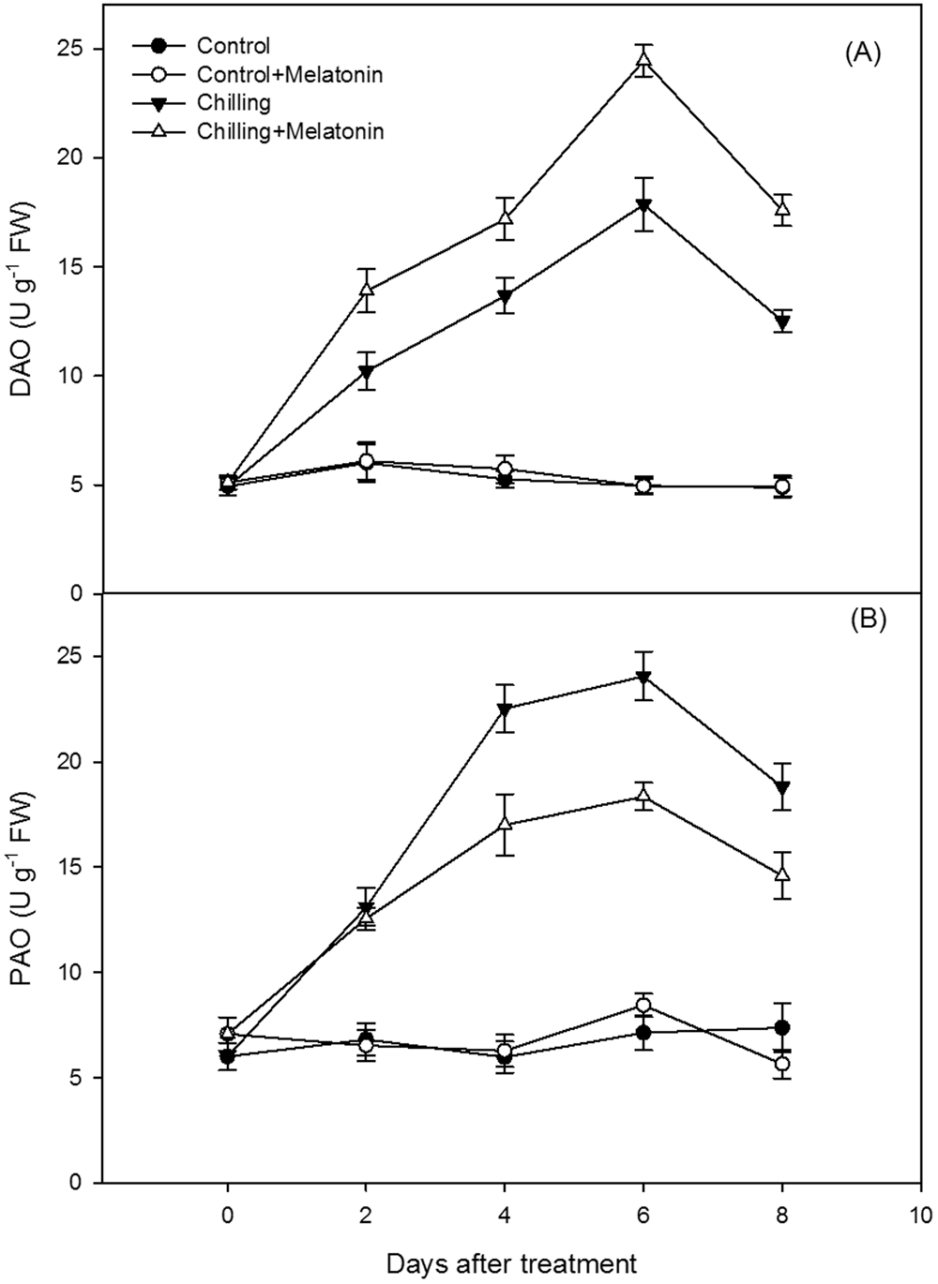
Effects of exogenous melatonin on polyamine degradation enzyme activities assay under chilling stress. (**A**) Diamine oxidase (DAO), (**B**) polyamine oxidase (PAO). Control, treating at 28/18 °C; Control + Melatonin, pre-treating with 200 μM melatonin and treating at 28/18 °C; Chilling, treating at 15/8 °C; Chilling + Melatonin, pre-treating with 200 μM melatonin and treating at 15/8 °C; Date represent means ± SD of third replicate samples.

Chilling increased DAO activity, levels continuing to rise until day 8 (Fig. 5A). The DAO activity response to chilling was significantly amplified by melatonin pretreatment. The PAO activity was also increased by chilling (Fig. 5B). Due to this increase of PAO activity, the degradation of Spm was accelerated, and Spm level of chilling treatment began decrease from day 4. However, melatonin had no significant effects on the PAO activity rise under chilling until day 4. After this, and through to day 8, the melatonin pretreatment significantly depressed the PAO activity response to chilling, which is helpful to maintain higher Spm content higher than chilling along.

### ABA content and transcriptional level of related genes

**Figure 6:**
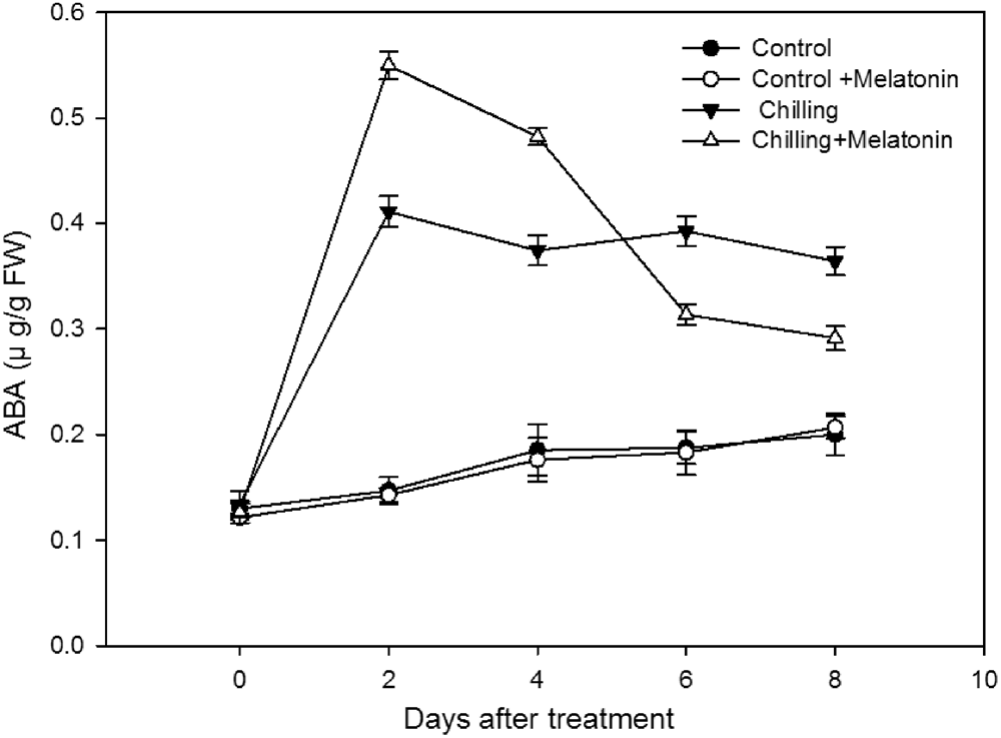
Effects of exogenous melatonin on abscisic acid (ABA) content under chilling stress. Control, treating at 28/18 °C; Control + Melatonin, pre-treating with 200 μM melatonin and treating at 28/18 °C; Chilling, treating at 15/8 °C; Chilling + Melatonin, pre-treating with 200 μM melatonin and treating at 15/8 °C; Date represent means ± SD of third replicate samples.

The ABA concentration rose dramatically over the first two days after chilling and then remained high over the next few days (Fig. 6). The melatonin pretreatment further increased ABA concentration over the first four days but then ABA declined from day 2, falling to levels lower than with no melatonin by days 6 and 8.

**Figure 7:**
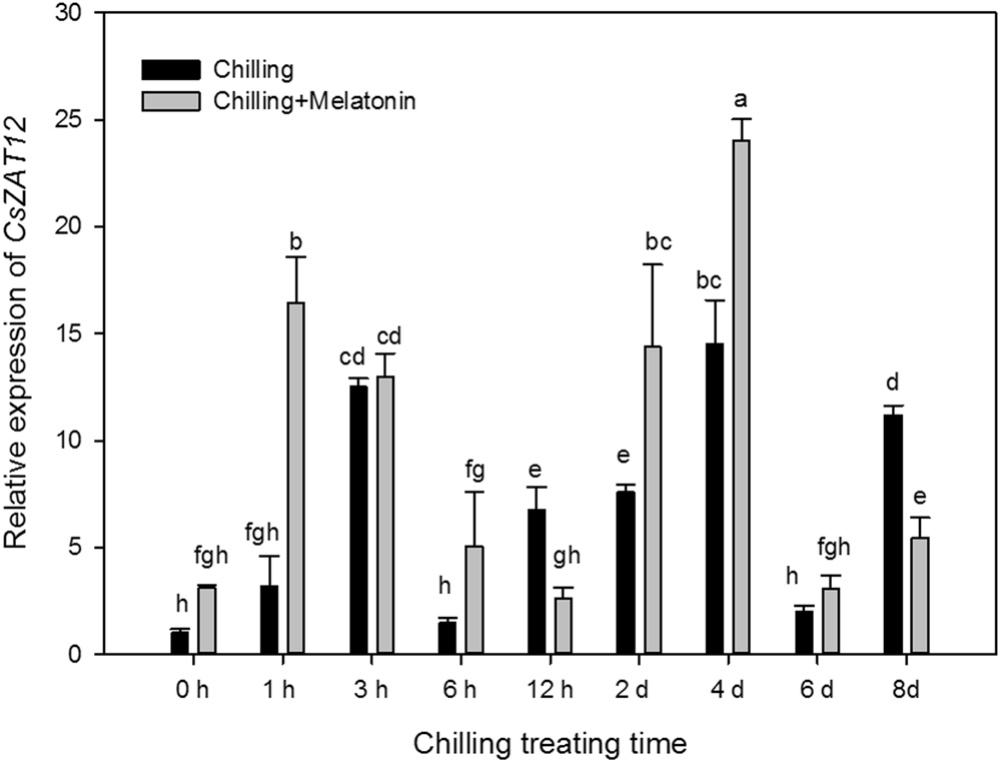
Effects of exogenous melatonin on transcript level of *CsZat12* under chilling stress. Control, treating at 28/18 °C; Control + Melatonin, pre-treating with 200 μM melatonin and treating at 28/18 °C; Chilling, treating at 15/8 °C; Chilling + Melatonin, pre-treating with 200 μM melatonin and treating at 15/8 °C; Date represent means ± SD of third replicate samples. Different letters indicate a significant difference in a particular series at P < 0.05 according to ANOVA and Duncan’s multiple range tests.

**Figure 8:**
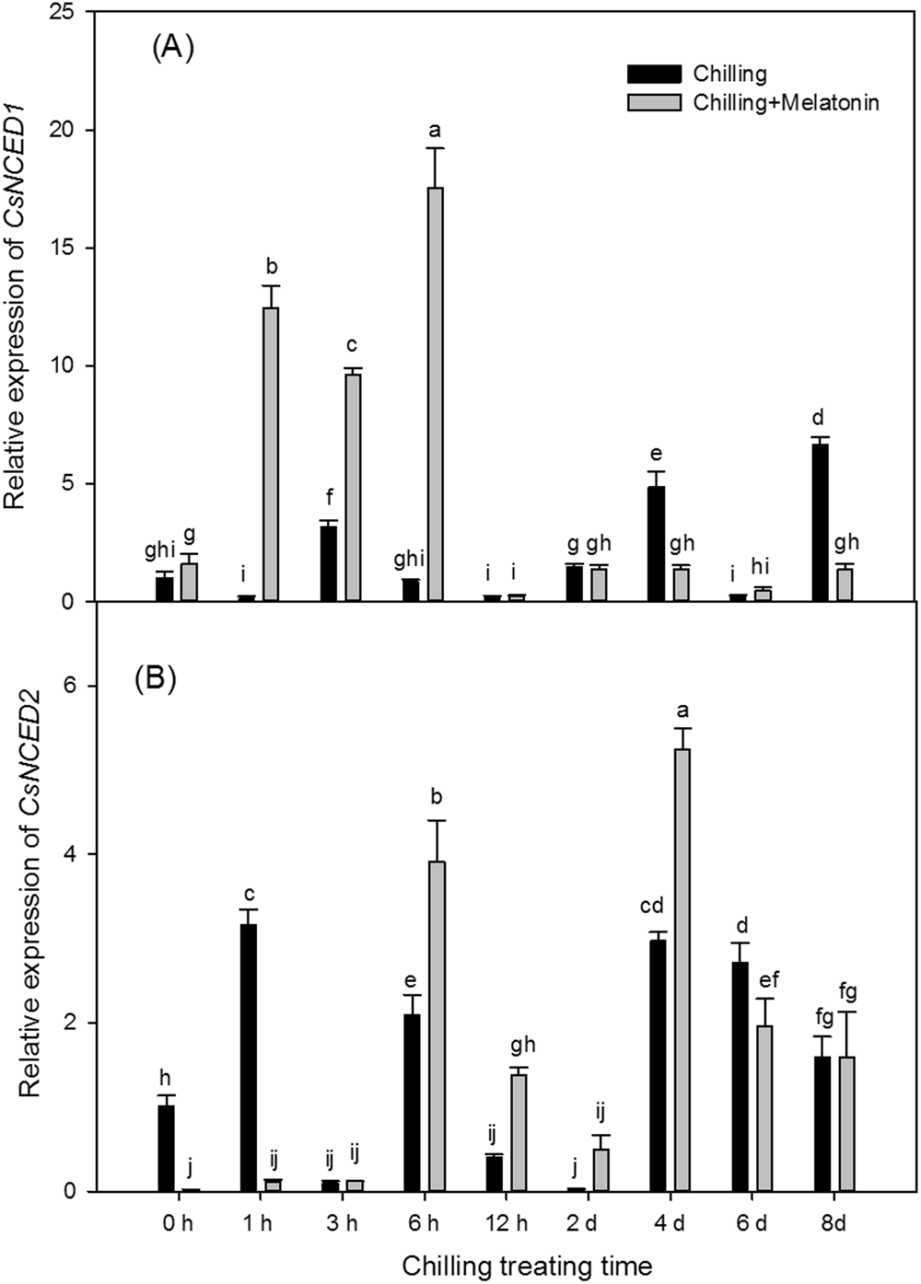
Effects of exogenous melatonin on transcript levels of key genes involved in biosynthesis of abscisic acid (ABA) under chilling stress. (**A**) *CsNCED1*, (**B**) *CsNCED2*. Control, treating at 28/18 °C; Control + Melatonin, pre-treating with 200 μM melatonin and treating at 28/18 °C; Chilling, treating at 15/8 °C; Chilling + Melatonin, pre-treating with 200 μM melatonin and treating at 15/8 °C; Date represent means ± SD of third replicate samples. Different letters indicate a significant difference in a particular series at P < 0.05 according to ANOVA and Duncan’s multiple range tests.

**Figure 9:**
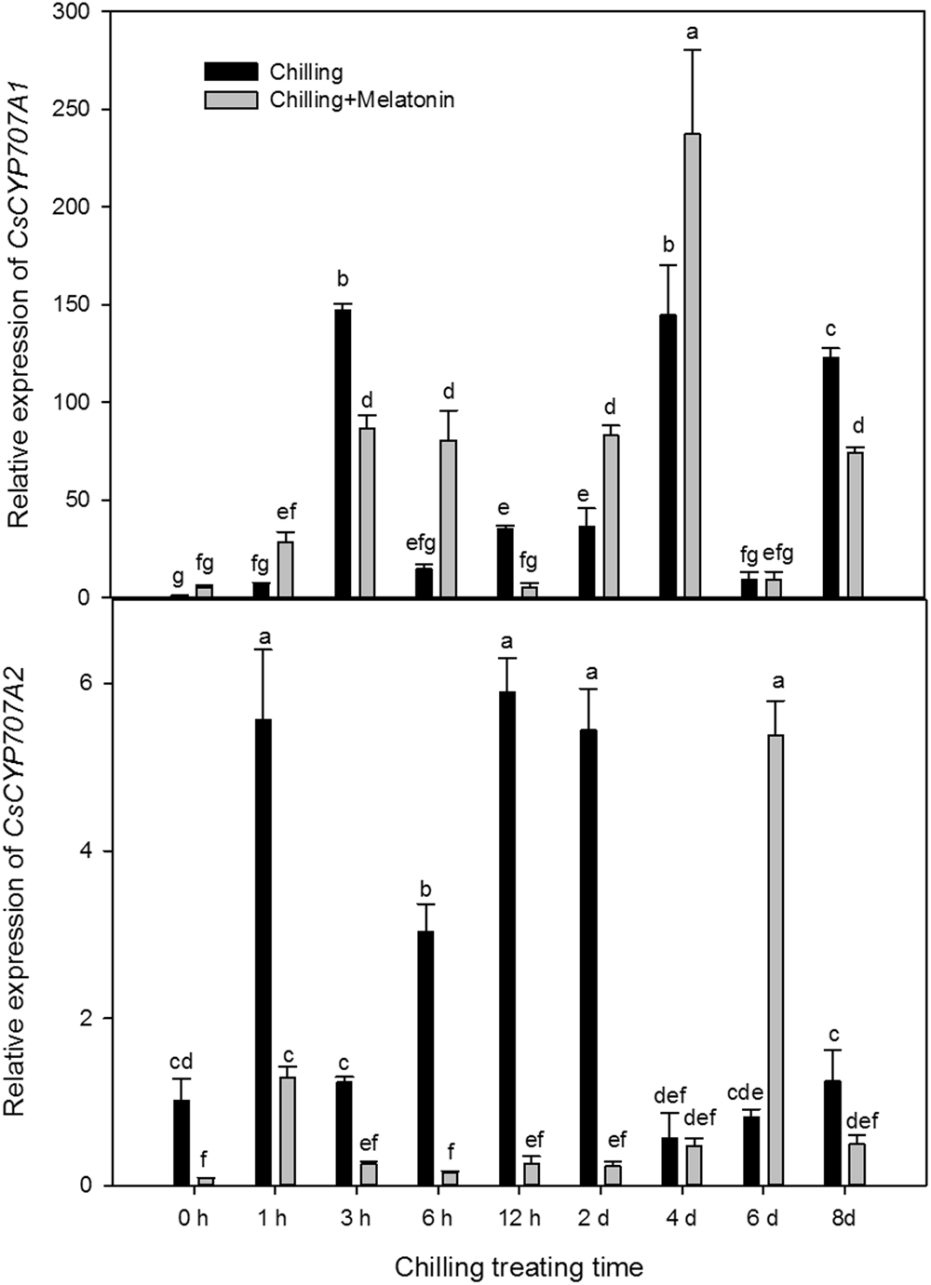
Effects of exogenous melatonin on transcript levels of key genes involved in catabolism of abscisic acid (ABA) under chilling stress. (**A**) *CsCYP707A1*, (**B**) *CsCYP707A2*. Control, treating at 28/18 °C; Control + Melatonin, pre-treating with 200 μM melatonin and treating at 28/18 °C; Chilling, treating at 15/8 °C; Chilling + Melatonin, pre-treating with 200 μM melatonin and treating at 15/8 °C; Date represent means ± SD of third replicate samples. Different letters indicate a significant difference in a particular series at P < 0.05 according to ANOVA and Duncan’s multiple range tests.

Expression levels of *CsZAT12* began to rise 3 h after chilling and generally remained high till day 8 (Fig. 7). Compared with the initial value of chilling treatment at 0 h, the express level of *CsZAT12* in chilling treatment was up-regulated by chilling 12.49-fold at 3 h, 14.50-fold at 4th day and 11.18-fold at 8th day. The melatonin pretreatment brought forward this up-regulation and also caused further up-regulation of *CsZAT12* at 1 h, 2 days and 4 days. Chilling did not significantly influence the expression of *CsNCED1* during the first 2 days but up-regulated it 3.19-fold at 3 h (Fig. 8A). However, *CsNCED1* expression was later up-regulated 4.86-fold at 4 days and 6.66-fold at 8 days. Melatonin dramatically up-regulated the expression of *CsNCED1* under chilling in the first 6 h but had no later effects. Chilling down-regulated the expression of *CsNCED2* over the first two days (except at 1 h and 6 h) but significantly up-regulated it over the next 6 days (Fig. 8B). Compared with chilling, melatonin further up-regulated *CsNCED2* from 6 h to 4 days but down-regulated it at 1 h. Compared with the expression level at 0 h, *CsCYP707A1* was dramatically up-regulated 147.31-fold at 3 h, 144.73-fold at 4 days and 122.77-fold 8 days (Fig. 9A). Compared chilling treatment, melatonin induced a marked rise in *CsCYP707A1* expression at 6 h, 2 days and 4 days but depressed it at 3 h and 8 days. After chilling, the expression of *CsCYP707A2* was up-regulated in the first 2 days but then fell to a level similar to that at 0 h over the next few days (Fig. 9B). Compared with chilling, melatonin significantly down-regulated the expression of *CsCYP707A2* over the first 2 days but up-regulated it at 6 days.

## Discussion

Chilling damage is a major factor limiting growth and development of cucumber. Under chilling, membrane fluidity decreases and enzyme activity reduces. These changes give rise to cell dysfunction^5^. The degree of membrane damage can be indicated by measuring the electrolyte leakage rate, because electrolyte leakage reflects membrane integrity^29^. Here, chilling induced a dramatic increase in electrolyte leakage but this response was significantly suppressed by melatonin pretreatment. Hence, it is reasonable to conclude that melatonin pretreatment can to some extent protect cucumber against chilling damage. To confirm this finding, we also monitored changes in Pn. Measurements of Pn offer another indicator of how the overall growth of a plant responds to chilling conditions. Here we show, Pn also declines dramatically under chilling, with the value at day 8 being only 19% of that at day 0 (Fig. 2). However, this response is significantly reduced in cucumber seedlings pretreated with melatonin. Such photosynthesis-preserving effects of melatonin have already been shown for cucumber under water stress^30^, for *Malus hupehensis* under salinity stress^31^ and for tomato under drought stress^32^. Consistent with these studies, our data confirm the mitigating effects of melatonin pretreatment on chilling stress in cucumber.

The gene *Zat12* encodes a zinc-finger transcription protein^24^. After first being isolated as a light-stress response cDNA (rhl41)^33^, *Zat 12* has since been found to respond to environmental stress from cold^34^, heavy metals^35^, and UV radiation^36^. As a key transcription factor involved in the activation of the ROS-related antioxidant system, *Zat12* accounts for up-regulation of about 8% of core cold-induced genes^22^, and plays an important role in enhancing plant tolerance to cold stress^37^. Here, chilling induced the over-expression of *CsZat12*, and melatonin further enhanced this over-expression (Fig. 7). Similar results have been found previously in *Arabidopsis thaliana* under chilling stress^12^. Our previous study (in review) found that under chilling, melatonin speeds up an important ROS-related antioxidant system, the ascorbate–glutathione (AsA-GSH) cycle. In this case, melatonin may activate *CsZat12* to stimulate this antioxidant system to protect plants from oxidative damage. In addition, *Zat12* is involved in the accumulation of Put through up-regulation of the expressions of *ADC1* and *ADC2* during cold stress^24^. This indicates that melatonin may regulate Put metabolism thus increasing chilling tolerance in plants.

Here, chilling induced significant increases in the contents of Put and Spd, and triggered a considerable variability in the level of Spm compared with the controls (Fig. 2). We observed that exogenous melatonin further increased the contents of Put and Spd and reduced the variability of Spm. It is apparent that these three PAs participate in the mechanism through which melatonin protects cucumber under chilling conditions.

**Figure 10:**
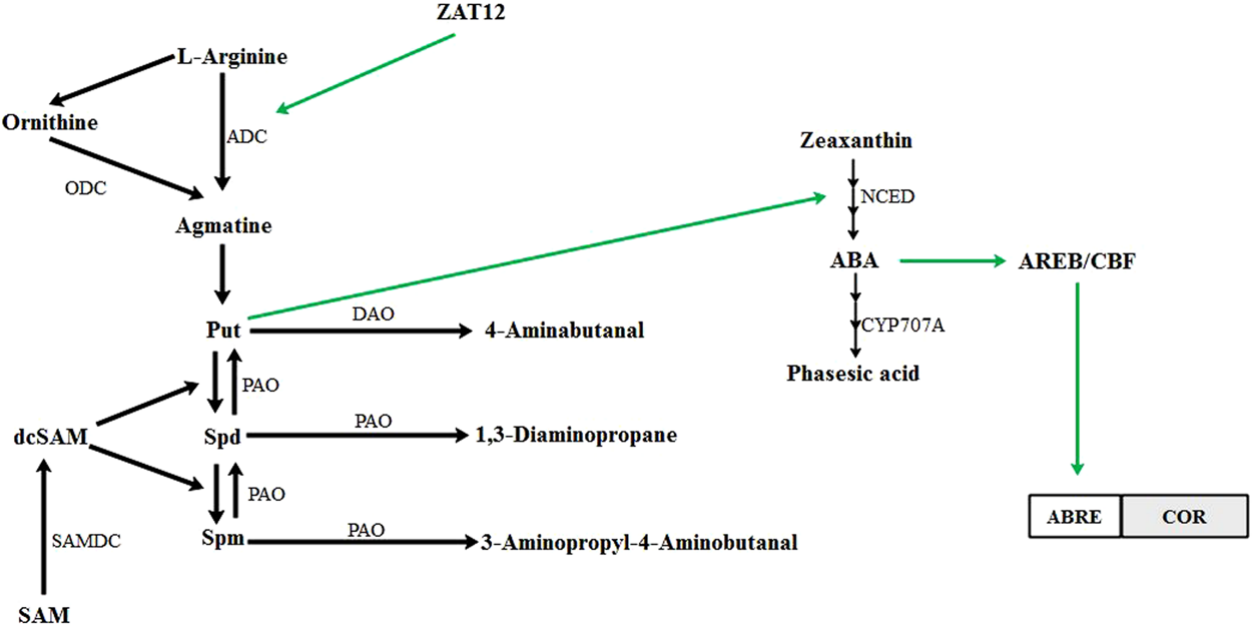
Metabolism of polyamines and abscisic acid (ABA) in plants. Put putrescine, Spd spermidine, Spm spermine, SAM S-adenosylmethionine, dcSAM decarboxylated S-adenosylmethionine, ADC, Arginine decarboxylase, ODC ornithine decarboxylase, SAMDC S-adenosylmethionine decarboxylase, DAO Diamine oxidase, PAO polyamine oxidase.

To reveal how melatonin affects PA levels, we investigated PA metabolism, which can be reflected by the activity of related enzymes. The three key enzymes synthesizing PAs are ADC, ODC and SAMDC, meanwhile DAO and PAO play important roles in PA catabolism (Fig. 10). In plants, Put is formed directly from arginine in a decarboxylase reaction catalyzed by ADC or ODC. Also, Spd and Spm are synthesized from Put in the following reactions with the addition of aminopropyl moieties arising from the decarboxylation of S-adenosylmethionine catalyzed by SAMDC^17^. Degradation of Put is consistent with DAO, whereas Spd and Spm degradations are catalyzed by PAO^38^. In our study, the activities of the PA synthesis enzymes, ADC, ODC and SAMDC and also those of the PA catabolism enzymes, DAO, PAO, were increased by chilling (Figs 4 and 5). It indicated that PA metabolism was enhanced by chilling. The strengthening of PAO activity induced the decrease of Spm in chilling treatment. Furthermore, melatonin further increased the activities of all the above enzymes except PAO. And, the suppression of melatonin on PAO activity was help maintain Spm content at a high level. It is suggested melatonin stimulates the activity of the PA metabolism enzymes thus influencing the content of PAs.

It has been confirmed that PAs are involved in the acquisition of plant tolerance to a range of different stresses^24^. Owing to their positive charge at physiological pH, PAs play an intrinsic structural role in the binding and stabilizing of negatively-charged macromolecules such as DNA and RNA, of proteins and of phospholipids^39^. In this way, they can also stabilize membrane structure, modulate enzyme activity and help keep nucleic acids stable, so as to enable the plant to cope with all kinds of stress^40, 41^. Our results confirm that melatonin can mitigate chilling stress by activating enzymes involved in the PA metabolism to increase the PA levels.

Increases in ABA are accompanied by increases in Put under cold stress. Also, Put content decreases when ABA synthesis is inhibited^42^. Furthermore, reductions in ABA caused by reduced *NCED3* expression can be restored by application of exogenous Put^43^. This finding suggests there may be an interaction between the PAs and ABA. This hypothesis has been confirmed in an increasing number of studies in recent years.

A previous study integrated the response of Put to low temperature and found that Put accumulation is closely related to ADC activation^44^. *Zat12*, a cold inducible C2H2 zing finger transcription factor, involves the upregulation of *ADC1* and *ADC2* ^22^. In turn, increases in Put activate the transformation from zeaxanthin to ABA (Fig. 10)^24^. The influence of ABA on PA metabolism has also been studied widely. It has been found that ABRE have been found in the promoters of genes such as *ADC2 and SAMDC1&2* which are the principle genes in PA metabolism^45^. It has been reported that the expression of *ADC2* can be impaired in ABA-deficient and ABA-insensitive *Arabidopsis* mutants^42^. In addition, the key PA catabolism genes, *PAO2, PAO3* and *PAO4*, can also be regulated by ABA^46^. Since there is clearly an interaction between ABA and the PAs, ABA may well be involved in the chilling tolerance increase caused by melatonin.

Based on the results reported here, chilling stress induces a dramatic increase in ABA content (Fig. 6). Similar results were found by Liu *et al*. in cucumber under chilling stress^47^. They suggest the increase in ABA level is just one part of the increase in chilling tolerance in cucumber. Moreover, melatonin significantly enhanced the increase over the first four days, although the ABA content declined significantly over the next few days (Fig. 6). This indicates melatonin plays a role in ABA metabolism. The modulation of melatonin on ABA metabolism has also been found in the process of germination in cucumber^48^. The difference from our results is that here, melatonin suppresses the rise in ABA after a short increase, compared with when melatonin is not applied during germination. The reason for this difference may be associated with the different growth stage. The decrease in ABA is necessary during germination in cucumber^48^ and whereas a high level of ABA in cucumber seedlings may help increase chilling tolerance^49^.

To gain further insight into these relationships, we monitored the time-course of the relative expressions of the ABA biosynthesis genes (*CsNCED1* and *CsNCED2*) and also of the ABA catabolism genes (*CsCYP707A1* and *CsCYP707A2*). These four genes were all up-regulated to varying degrees by chilling (Figs 7 and 8). The high expression level of the ABA biosynthesis genes (*CsNCED1* and *CsNCED2*) is related to the increase of Put level, which is consist with the previous study^23, 24^. Also, ABA level increased over the first two days (Fig. 6). This suggests ABA biosynthesis may play a leading role in ABA catabolism. In the two ABA catabolism genes, only the expression of *CsCYP707A1* was up-regulated, whereas the expressions of both the ABA biosynthesis genes stayed high over the following six days of chilling. This indicates the fluctuation of ABA over the last six days, following two days of significant increase, is due mainly to *CsCYP707A1*. Moreover, *CsCYP707A1* was further up-regulated by melatonin at days 2 and 4, which caused an obvious decline in ABA after day 2. The dramatic increase in ABA content over the first two days chilling treatment in cucumbers pretreated with melatonin was related to *CsNCED1* and *CsCYP707A2*, as melatonin significantly up-regulated *CsNCED1* but suppressed *CsCYP707A2* compared with that in chilling along treatment in the first two days. These results suggest melatonin makes a significant contribution to the levels of ABA by regulating the expression of the ABA metabolism genes.

It is well known ABA controls many stress adaption responses by activating genes involved in resistance to adverse conditions. Although ABA acts mainly through ABA-dependent pathways, such as when induced by osmosis stress, there is an overlap in the expression pattern of stress genes between chilling and osmosis^49^. As a result, ABA may induce the expression of stress genes so as to enhance chilling tolerance. This has been confirmed in potato, treated with exogenous ABA^50^. Apart from this, there is also cross-talk between the ABA-dependent pathway and the ABA-independent pathway^51^. For example, both drought responsive element (DRE) and ABA- responsive element (ABRE) are found in the *RD29A* promoter^52^. As stress tolerance is acquired mainly through the ABA-dependent and ABA-independent pathways, ABA may play an important role in the mitigation of chilling injury induced by application of exogenous melatonin.

Melatonin has a significant involvement in mitigating chilling stress in cucumber. The mechanisms though which melatonin alleviates chilling injury may be presented as follows. First, melatonin up-regulates *CsZat12*, an important stress related gene. Second, melatonin increases PA content by enhancing the activity of the PA metabolism enzymes. And, third, ABA is involved in these effects as melatonin modulates the expression of the genes regulating ABA metabolism. This study provides new evidence suggesting that melatonin mitigates chilling stress in cucumber by up-regulating the expression of *CsZat12* and by modulating the metabolism of PAs and of ABA.

## Methods

### Plant material and treatments

Seeds of cucumber (*Cucumis sativus* L.) were sown in black plastic pots (7 × 7 cm) filled with a mixed nutrient medium and placed in a growth chamber with a normal growing environment (28/18 °C day/night, 75% relative humidity, 300 μmol·m^−2^·s^−1^ photon flux density and 12 h photoperiod). When the second true leaf was fully expanded, half the seedlings were pretreated by irrigation with a 200 μM melatonin, solution while the rest were irrigated with distilled water. The two pretreatment irrigations were maintained until the experiment was concluded. Five days after the pretreatment, the two groups of pretreated seedlings were each randomly divided in half. One half of each of the two groups were moved to another growth chamber operating under the same general environmental conditions but at chilling temperatures (15/8 °C day/night) while the other half grew on in the original growth chamber under non-chilling conditions (28/18 °C day/night). This protocol resulted in four experimental groups of plants: (i) Control + Water: grown at normal temperatures, irrigated with distilled water; (ii) Control + Melatonin: grown at normal temperatures, irrigated with melatonin; (iii) Chilling + Water: grown at chilling temperatures, irrigated with distilled water; (iv) Chilling + Melatonin: grown at chilling temperatures, irrigated with melatonin.

Leaf samplings were taken at increasing time intervals after the imposition of chilling, at 0, 1, 3, 6 and 12 h and at 2, 4, 6 and 8 d. These were immediately frozen in liquid nitrogen and stored at −80 °C pending analysis. Photosynthetic parameters were measured on intact plants at 0, 2, 4, 6 and 8 d after chilling.

### Electrolyte leakage analysis

Twenty, freshly-cut leaf discs (0.5 cm diameter) were incubated in 10 mL deionized water at 25 °C. The electrical conductivity of the incubating solution (E1) was measured after 3 h using a DDSJ-308A conductivity meter (Leici, China). The samples were then boiled in their incubation solution for 10 min. After cooling, the solution’s electrical conductivity (E2) was again measured. Results are expressed as the percentage of electrolyte leakage (%) in deionized water (E1/E2 × 100).

### Measurement of photosynthetic rates

Plants were moved to a growth chamber at 25 °C, and maintained under a photon flux density of 600 μmol·m^−2^·s^−1^ for 1 h before measurement. Leaf gas exchange parameters were measured simultaneously using a LI-6400XT equipped with a leaf chamber fluorimeter LI6400-40 (LI-COR, Lincoln, NE, USA). The air temperature, CO_2_ concentration and photosynthetic photon flux intensity (PPFD) in the leaf chamber were set at 25 °C, 350 μmol·mol^−1^ and 600 μmol·m^−2^ s^−1^, respectively.

### Polyamine isolation, identification and analysis

Polyamines were determined according to the method of Hu *et al*., with slight modification^53^. Leaves (1 g) were extracted in 5% cold perchloric acid (PCA) for 1 h. The extract was centrifuged at 12, 000 × g for 30 min. The supernatants (1 mL) were mixed with 2 mL of 2 M NaOH and 10 μL of benzoyl chloride. The benzoylation action was terminated using 4 mL of saturated NaCl solution after 30 min at 37 °C. The benzoyl PAs were extracted with 3 mL of diethyl ether by centrifuging at 3000 × g for 5 min. A volume of 2 mL of the ether phase was evaporated to dryness and the residue re-dissolved in 500 μL methanol. Polyamines were assayed by high-performance liquid chromatography (LC-20A Prominence, Shimadzu Co. Ltd., Japan). A volume of 10 µL of the benzoyl PAs in methanol were injected into a 20 mL loop, loaded onto a 4.6 × 250 mm, 5 mm particle size, reverse phase Kromasil C18 column (Eka Chemicals, Bohus, Sweden). Column temperature was maintained at 25 °C. Samples were eluted from the column with 64% methanol at a flow rate of 0.7 mL min^−1^ using a Dionex P680 pump. Polyamine peaks were detected with a UV detector at 254 nm.

### PA biosynthesis enzyme activity assay

Leaves (0.5 g) were extracted in 100 mM potassium phosphate buffer (pH 8.0) containing 0.1 mM phenylmethylsulfonyl fluoride (PMSF), 1 mM pyridoxal phosphate (PLP), 5 mM dithiothreitol (DTT), 5 mM EDTA, 25 mM ascorbic acid, and 0.1% polyvinylpyrrolidone (PVP). The extract was centrifuged at 12,000 × *g* for 40 min at 4 °C. The supernatants were dialyzed at 4 °C against 3 mL of 100 mM potassium phosphate buffer (pH 8.0) containing 0.05 mM PLP, 1 mM DTT, and 0.1 mM EDTA for 24 h in darkness. The dialyzed extract was used in the enzyme assay.

The activities of arginine decarboxylase (ADC; EC 4.1.1.19), ornithine decarboxylase (ODC; 4.1.1.17) and S-adenosylmethionine decarboxylase (SAMDC; EC4.1.1.50) were determined as described by Zhao *et al*. and Hu *et al*. with some modifications^54, 55^. 0.3 mL of the dialyzed enzyme extract was mixed with 1 mL of the reaction mixtures containing 100 mM Tris-HCl buffer (pH 7.5), 5 mM EDTA, 50 mM pyridoxal phosphate, 5 mM DTT. After which 0.2 mL of 25 mM L-arginine, L-ornithine or S-adenosylmethinonine was added respectively, The mixtures were incubated at 37 °C for 1 h. After that, PCA was added until the final concentration of PCA was 5% (PCA was added prior to the 1 h incubation for the positive control). After centrifuging at 3000 × *g* for 10 min, 0.5 ml of the supernatant was mixed with 1 mL of 2 mM NaOH and 10 μL benzoyl chloride, and then stirred for 20 s. After incubation for another 30 min at 37 °C, the mixture was added 4 ml saturated NaCl solution and 3 ml ether. 2 ml of the ether phase was evaporated to dryness and re-dissolved in 1 ml 60% methyl alcohol. The absorbance of this solution was measured at 254 nm using a Shinadzu UV1800 specrophotmeter (Shimadzu Co. Ltd; Japan). Enzyme activities were expressed in nmol Arg g^−1^ FW h^−1^, nmol Put g^−1^ FW h^−1^ and nmol SAM g^−1^ FW h^−1^.

### PA degradation enzyme activity assay

Activities of diamine oxidase (DAO; EC 1.4.3.6) and polyamine oxidase (PAO; EC 1.5.3.3) were monitored as described by Hu *et al*.^52^. Leaves (0.5 g) were extracted in 100 mM potassium phosphate buffer (pH 6.5). After centrifuging at 10,000 × *g* for 20 min at 4 °C, 0.2 mL of the supernatant was mixed with 2.8 mL potassium phosphate buffer (100 mM, pH 6.5), 0.2 mL 4-aminoantipyrine/N, N-dimethylaniline reaction solution and 0.1 mL horseradish peroxidase (250 units mL^−1^). Reaction were initiated by the addition of 15 μL of Put or Spd to a final concentration of 20 mM respectively. A unit of enzyme activity was defined as one giving a change in optical density of 0.001 absorbance units at 254 nm.

### Determination of ABA content

Leaves (0.1 g) were ground with a ball mill in Eppendorf vials, to which 1 mL of cold 80% methanol was then added. After incubation at −20 °C for 12 h, the extraction was centrifuged at 8000 × g for 30 min. The supernatant was purified with a C-18 solid-phase extraction column. The purified solution was dried in a vacuum concentrator and then re-dissolved in 1 mL of sodium phosphate buffer (pH 7.4, 10 mM). The ABA content was determined with enzyme-linked immunosorbent assay (ELISA) kit (R&D systems USA) according the manufacturer’s instructions.

### RNA isolation and quantitative real-time RT-PCR

Total RNA was extracted from leaves with a plant RNA kit (Omega Bio-Tek, Doraville, GA, USA) and then reverse-transcribed using a PrimeScript^TM^ RT reagent kit with gDNA Eraser (Takara, Shiga, Japan) according to the manufacturer’s instructions^14^. Real-time PCR was carried out on a FTC-3000p real-time PCR cycle (Funglyn Biotech, Canada) using a SYBR Premix EX Taq kit (Taraka). The specific primers (Table S1) used were designed using Primer Premier 6 software (Biosoft International, Palo Alto, CA, USA). The actin gene acted as the internal standard.

### Statistical analyses

The data were expressed as the means ± one standard deviation of three replicate samples. One way ANOVA was used to analyze all data, followed by Duncan’s multiple range tests. A value with P < 0.05 was considered statistically significant.

## Electronic supplementary material

Supplementary information
