# Supplementary material for: Melatonin alleviates chilling stress in cucumber seedlings by up-regulation of CsZat12 and modulation of polyamine and abscisic acid metabolism
Source: Sci Rep. 2017 Jul 10;7:4998. doi: 10.1038/s41598-017-05267-3 (PMC5504047; doi:10.1038/s41598-017-05267-3)
Supplement: Supplementary file 1 — Supplementary information [file 41598_2017_5267_MOESM1_ESM.pdf]

Melatonin alleviates chilling stress in cucumber seedlings by up-regulation of *CsZat12* and modulation of polyamine and abscisic acid metabolism

Full name of author(s): Hailiang Zhao [1, 2,3+*], Kai Zhang[1, 3+], Xiaoting Zhou [1, 3+], Linjie Xi [1, 3], Yuping Wang[4], Hongjun Xu [1, 3], Tonghua Pan [1, 3] and Zhirong Zou [1, 3]

Institution of affiliation: 1 College of Horticulture, Northwest Agricultural & Forest University, Yangling, Shaanxi, China, 2 College of Horticulture, Shanxi Agricultural & Forest University, Taigu, Shanxi, China 3 Key Laboratory of Protected Horticulture Engineering in Northwest, ministry of Agriculture, Yangling, Shaanxi, China, 4 Department of Garden Engineering, Gansu Agriculture Technology College, Lanzhou, Gansu, China.

+These authors contributed equally to this work.

***Corresponding author:**

Hailiang Zhao

Hailiang127627@126.com

Supporting Table 1. Sequence of primers used in quantitative real-time RT-PCR.

Gene	Accession NO.	Primer Sequence (5’-3’)
actin	Csa018882	F: ATGGCCGATGCCGAGGATAT
		R: TAGGAGCATCATCACCAGCAAAAC
CsZat12	Csa5M180920.1	F: TGCAATCGGCAGTTTTCGTC
		R: TGCGTCTTCGGCTTAGTAGG
CsNCED1	Csa4M064690.1	F: GGAAGTGGTGGTGATTGGGT
		R: GTCGATTTCCCCGTCCGTAA
CsNCED2	Csa7M428120.2	F: CAAATCCGAAGTTTAGCCCAG
		R: CATAATCCAGCAGACCAAGCG
CsCYP707A1	Csa4M056600.1	F: TCGATGCCGATTAACCTCCC
		R: CCGCTTCGTTTCCCTTCTTG
CSCYP707A2	Csa2M357280.1	F: TCGGAGTTCTGTTTGCGGCT
		R: TGGTAAAGGGCATAGTTCGT
